# Variation in post-operative weight-bearing practice following hip fracture surgery: A national hip fracture audit review

**DOI:** 10.1016/j.jcot.2025.103200

**Published:** 2025-09-05

**Authors:** Helen Smith, Amy Lindh, Alex Aquilina, Alex Trompeter, Antony Johansen, Will Eardley

**Affiliations:** a South Tees Hospitals NHS Foundation Trust, Middlesbrough, UK; b National Falls and Fragility Fracture Audit Programme (FFFAP), Royal College of Physicians, London, UK; c Department of Health Sciences, University of York, York, UK; d St George's University Hospital, London, UK; e St George's University of London, London, UK; f Musculoskeletal Research Unit, Translational Health Sciences, Bristol Medical School, University of Bristol, Bristol, UK; g University Hospital of Wales, Cardiff, UK; h School of Medicine, Cardiff University, Cardiff, UK

**Keywords:** Hip fracture, Weight bearing, Mobilisation, NHFD, Elderly trauma

## Abstract

**Background:**

Hip fracture care has undergone a significant transformation, with a growing emphasis on patient experience and functional outcomes. Following national guidance that recommends early unrestricted weight-bearing, it is assumed that this is standard practice across all trauma units.

**Methods:**

Using anonymous aggregate data from the National Hip Fracture Database (NHFD) for the 2023 calendar year, weight-bearing status for patients who underwent primary hip fracture surgery across 169 hospitals in England, Wales, and Northern Ireland was investigated. The study excluded revision surgeries, periprosthetic fractures, non-operative cases, and patients who died before surgery.

**Results:**

Of 74,513 patients studied, 93.2% were fully weight-bearing, 3.1% were non-weight-bearing, and 3.6% had no data recorded. Significant variation existed across hospitals, with nine hospitals reporting 10% or more of their hip fracture cases as non-weight-bearing and one site recording 100% of patients as non-weight-bearing.

**Discussion:**

This study challenges the assumption of uniform early weight-bearing practices. With projected increases in hip fracture admissions and associated healthcare costs, optimising patient mobilisation is crucial. The wide variation in weight-bearing instructions suggests inconsistent application of clinical guidance, which will likely impact patient recovery, length of stay, and overall healthcare efficiency.

**Conclusion:**

Mobilisation after hip fracture surgery needs to be more consistent. Healthcare leaders must remain vigilant in ensuring full weight-bearing becomes the default approach and seek justification where this is not the case.

## Key Highlights

• Only 93.8 % of hip fracture patients were allowed to bear full weight in 2023, contrary to the assumed standard practice.
• High variation exists across hospitals, with some units reporting that 10 % or more of their patients are non-weight-bearing.
• Early weight-bearing is crucial for patient recovery and may help alleviate the growing burden of hip fractures.
• This study calls for standardised approaches to post-surgical mobilisation across all trauma units.

## Graphical abstract

**Figure:**
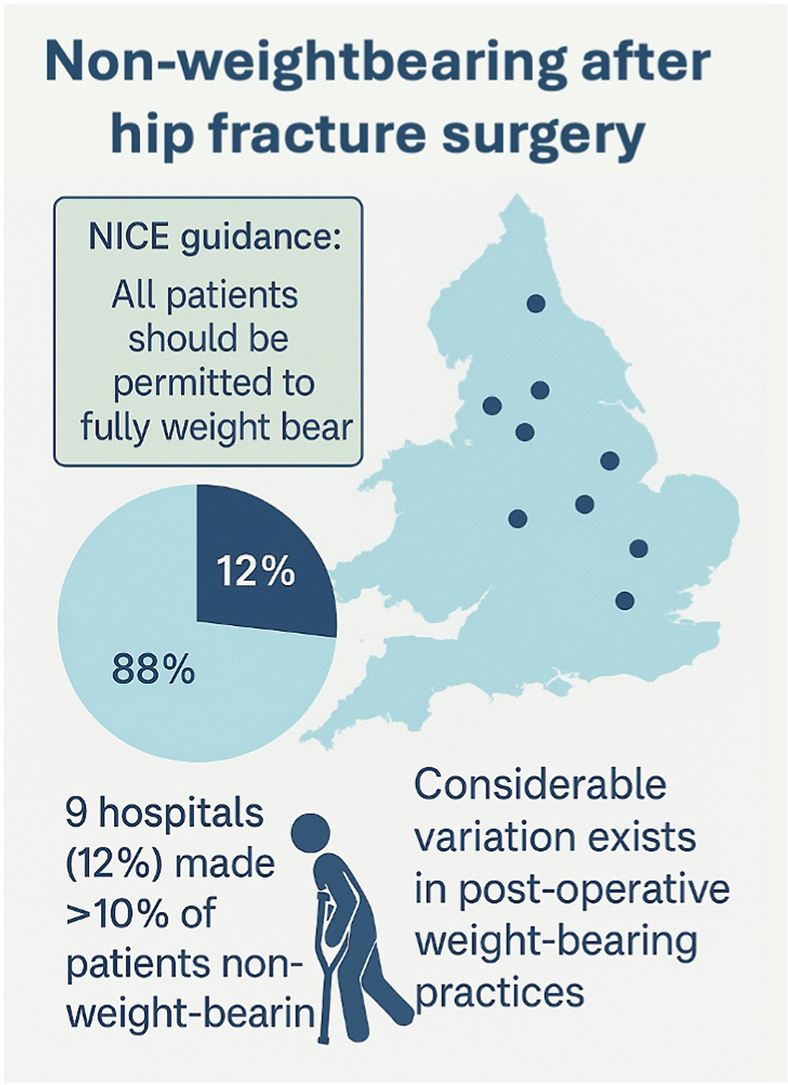

## 1 Introduction

The National Hip Fracture Database (NHFD) collects data on every patient presenting with a hip fracture in England, Wales and Northern Ireland. The NHFD reports Key Performance Indicators (KPIs) designed to assess and improve care. The number of KPIs collected on operative (implant-related and surgical process) interventions is minimal. KPIs, measuring preoperative care, cohorting of patients and orthogeriatric review, have a more significant impact on the patient experience and are therefore more critical to the overall patient outcome^2^. The World Hip Trauma Evaluation (WHiTE) Study's portfolio demonstrates this change in research focus, with studies on pressure lesions and physiotherapy input now recruiting^1^^,^^3^, ^4^, ^5^, ^6^, ^7^. As the number of hip fractures increases, so does the need to improve care effectiveness^8^^,^^9^.

One aspect of this cultural shift, focusing on patient function and experience, is simply getting out of bed. Increasing physiotherapy input to patients with a broken hip is beneficial but only effective in surgical practice, where instruction for early unrestricted weight-bearing is the standard, as recommended by the NICE hip fracture clinical guideline in 2011^10^. Hip fracture patient care exemplifies how this should be done, and it is increasingly recognised that a standard of early weight-bearing will likely benefit all patients with non-ambulatory fragility fractures (NAFF)^11^^,^^12^.

Trompeter's “Call to Arms” in 2020^13^ has been echoed by an increasing number of orthopaedic surgeons in the UK who recognise the importance of early weight-bearing and mobilisation, particularly in older patients^12^^,^^14^. As a result, informal conversations with surgeons can lead to the impression that all hip fracture patients across the UK are routinely made fully weight-bearing by default.

This study tests this assumption, using NHFD data to examine whether immediate unrestricted weight-bearing is the default in all patients following surgery for hip fracture.

## 2 Methods

The NHFD is a mandatory national clinical audit built to facilitate improvements in the quality of hip fracture care. It is a mature data collection system that, over 17 years, has captured over 95% of all patients aged 60 and above presenting to 174 trauma units in England, Wales, and Northern Ireland ^8^^,^^9^.

Anonymous aggregate data released by the NHFD as a routine part of its reporting for the 2023 calendar year (www.nhfd.co.uk/20/hipfractureR.nsf/docs/2024Report) was used in the analysis. This includes any patient receiving primary surgery for a hip fracture and excludes people having revision hip fracture surgery. We also excluded surgery for a periprosthetic fracture, patients who died before surgery was possible, and those managed non-operatively. Weight-bearing status is recorded by local clinical teams based on immediate post-operative instructions, typically documented in operative notes. As the NHFD collects this as part of its routine dataset, definitions and thresholds may vary slightly between units. However, the overarching categorisation is into ‘full weight-bearing’ or ‘non-weight-bearing’ as determined by the surgical team.

We included sites with missing data and have reported the extent of this. Descriptive analysis was used to calculate mean values for all sites included (Microsoft® Excel® for Microsoft 365 MSO (Version 2411 Build 16.0.18227.20082) 64-bit). The analysis was based on the complete national dataset from the NHFD for the 2023 calendar year; no sampling or inferential statistics were required, and therefore, assumptions about data normality were not applicable.

This study used fully anonymised, publicly available data from the National Hip Fracture Database and did not require ethical approval. Institutional Review Board approval was not required for this study.

## 3 Results

In 2023, 169 hospitals reported the weight-bearing status of 74,513 patients following surgery for hip fracture. Overall, 93.2% were made ‘full weight-bearing’, 3.1% (2310) non-weight-bearing, and the remaining 3.6% did not have data recorded.

**Fig. 1:**
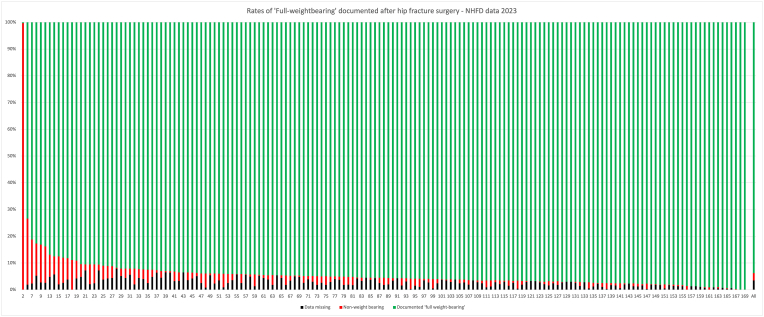
Rates of 'Full Weight-Bearing' documented after hip fracture surgery. National Hip Fracture Database 2023 data presented.

Fig. 1 depicts this but also shows the variation across units in the frequency of non-weight-bearing. The full table is available in the supplementary materials. Nine hospitals reported making 10% or more of their hip fracture cases non-weight-bearing – one site recorded 100% as non-weight-bearing.

## 4 Discussion

The hip fracture population is growing, and any factor impacting the effectiveness of care is important and deserves focus. Within four years, the total annual bed occupancy for hip fracture patients in Scotland is anticipated to increase by over 60,000 bed days, costing an additional £25 million. This increase equates to five additional hip fracture beds per hospital^15^.

In England and Wales, the effect of demographic change is projected to show that current admissions will increase by one quarter by 2030 and double by 2060^8^. This will result in hospitals increasingly struggling to care for frail, comorbid patients at increased risk of postoperative complications, who are likely to require additional health and social care support^16^, ^17^, ^18^, ^19^.

To maintain current standards of care in the face of this trajectory, the efficiency of hip fracture care will have to increase; any measure impacting Length of Stay (LOS) and patient mobilisation must be identified and optimised^2^. The anecdotally assumed default expectation of full weight-bearing for all hip fracture patients is one such measure thought to improve matters.

By reporting wide variation in weight-bearing status across hospitals in England, Wales and Northern Ireland, this study demonstrates that this assumption does not hold. Our findings are of concern, given that early mobilisation is associated with reduced LOS, 30-day mortality, and complication rates^20^^,^^21^. A culture of early weight-bearing may seem standard for hip fracture care, but considerable variation exists across units. Many units are still making older frail patients non-weight-bearing, and several record more than 10 % of patients as being left non-weight-bearing after surgery.

This variation is unlikely to reflect patient-specific contraindications alone and may instead stem from entrenched local practices, surgeon preference, or institutional habit. Historical surgical training, variation in physiotherapy access, and differing interpretations of implant stability likely contribute. That only certain units consistently restrict weight-bearing suggests regional cultural differences in practice rather than evidence-based decision-making.

Studies show a significant loss of knee extensor strength and stair climbing power after 10 days of bed rest in healthy volunteers^22^. Additionally, restricted weight-bearing in older patients requires significantly more energy expenditure than normal walking^23^. Therefore, the surgeon should not view such limitations as benign prescriptions, regardless of the injury or operative intervention.

The observations presented in this study are significant because, as a community, if we are not getting weight-bearing right in the context of hip fracture, despite clear guidance and well-established recovery benefits, how can we hope to translate these benefits to other patients following NAFFs? We also argue that lessons learned in the non-surgical elements of hip fracture patient care should be applied to the frail trauma patient population as a whole.

Since 1990, the average age of trauma patients has increased by 30 years^24^. The majority of major trauma now occurs in older adults falling from less than 2 metres, and adults aged over 60 constitute over half of those who undergo fracture fixation^25^. These injuries should be managed according to the same principles as hip fractures, with early intervention, mobilisation and orthogeriatric assessment^12^^,^^26^. Only a third of lower limb fragility fractures are allowed full weight bearing after operative intervention^14^.

Creating, changing and promoting wider care pathways to optimise weight-bearing following NAFFs likely represents low-hanging fruit for cost and efficiency savings in NHS trauma care. Therefore, future research into weight-bearing after surgery for fragility fractures should consider the broader financial implications for health and social care systems.

A recent consensus statement and guidance have been published to help standardise weight-bearing terminology and encourage clinicians to carefully consider the implications of weight-bearing^27^^,^^28^. This seeks to encourage a move towards ‘default unrestricted weight-bearing’.

There are limitations to this work. The audit does not record why patients were instructed not to weight-bear, and, as with any national audit dataset, we cannot infer causality. Data is missing, albeit minimal, which does not affect the proportionality of the results. While this study does not directly link weight-bearing status to outcomes such as mortality or implant failure, this relationship is well-established in the literature. Early mobilisation and unrestricted weight-bearing are associated with improved outcomes, including reduced 30-day mortality, fewer complications, and shorter hospital stays, making additional linkage analyses unnecessary to support this standard of care^11^, ^12^, ^13^^,^^20^^,^^21^^,^^23^^,^^27^^,^^28^. Publicly available NHFD data did not allow us to examine the relationship between post-operative weight-bearing status and implant use, age, or other demographic factors. However, the NHFD collects such data, and this may present an opportunity for future detailed analysis.

## 5 Conclusion

Hip fractures are a key focus for healthcare improvement for patients, as well as for healthcare services facing a growing burden of admissions. Until now, there has been an anecdotal presumption that full weight-bearing is routine. This assumption has led to a focus on other injuries in older patients. Our findings suggest that this shift in focus is premature.

Those managing and leading hip fracture care locally should remain vigilant to ensure all are made full-weight-bearing post-surgery and seek justification where this is not the case. Mobilisation after hip fracture surgery remains a work in progress.

## Patient consent statement

Patient consent was not required for this study as it utilised anonymised aggregate data that is openly available in the public domain through the National Hip Fracture Database (NHFD) website.

## Ethics statement

The National Hip Fracture Database (NHFD) operates as part of the Falls and Fragility Fracture Audit Programme (FFFAP) under the governance of the Healthcare Quality Improvement Partnership (HQIP). The audit's ethical framework is established through Section 251 approval from the Health Research Authority's Confidentiality Advisory Group (CAG).

This approval, granted under Regulation 5 of the Health Service (Control of Patient Information) Regulations 2002, permits collecting and processing confidential patient information without explicit consent. The specific CAG reference for NHFD data collection is CAG 8-03(PR11)/2013.

The data collection process adheres to NHS Digital's Data Security and Protection Toolkit requirements, ensuring compliance with national data protection standards. Healthcare providers submit data to the NHFD as part of their NHS Standard Contract obligations, which mandates participation in relevant national clinical audits.

For publication and research purposes, all data is anonymised in accordance with the Information Commissioner's Office Anonymisation Code of Practice. The open-access publication of findings falls under the audit's core purpose of improving patient care through transparency and shared learning and does not require additional ethical approval as it uses exclusively anonymised data.

The Royal College of Physicians, as the host organisation for the FFFAP, maintains oversight of the audit's data governance processes and ensures compliance with all relevant regulatory requirements.

This study used fully anonymized, publicly available data from the National Hip Fracture Database and did not require ethical approval.

## Author contributions (CRediT taxonomy)

**Helen Smith:** Data curation; Formal analysis; Writing – original draft.

**Amy Lindh:** Data curation; Formal analysis; Writing – original draft.

**William Eardley:** Conceptualisation; Supervision; Writing – original draft.

**Antony Johansen:** Conceptualisation; Supervision; Writing – original draft.

**Alex Trompeter:** Conceptualisation; Supervision; Writing – original draft.

**Alex Aquilina:** Writing – review & editing.

All authors reviewed and approved the final manuscript.

## Ethical statement

This study used fully anonymised, publicly available data from the National Hip Fracture Database and did not require ethical approval.

## Funding statement

This project received no specific grant from any funding agency in the public, commercial, or not-for-profit sectors. William Eardley and Antony Johansen received institutional funding through the Royal College of Physicians’ Falls and Fragility Fracture Audit Programme (FFFAP) in their roles as clinical leads for the National Hip Fracture Database (NHFD).

## Declaration of interest statement

On behalf of all authors (Helen Smith, Amy Lindh, Alex Trompeter, Alex Aquilina, Antony Johansen, and Will Eardley), I declare that we have no known competing financial interests or personal relationships that could have appeared to influence the work reported in this paper.
