# Supplementary material for: Variation in post-operative weight-bearing practice following hip fracture surgery: A national hip fracture audit review
Source: J Clin Orthop Trauma. 2025 Sep 5;70:103200. doi: 10.1016/j.jcot.2025.103200 (PMC12495072; doi:10.1016/j.jcot.2025.103200)
Supplement: Multimedia component 1 [file mmc1.docx]

**Supplementary Material 1. A table of 2023 NHFD data on documented weightbearing status after hip fracture surgery (Anonymised)**

| **Site** | **Data missing** | **Non-weight bearing** | **Documented 'full weight-bearing'** |
| --- | --- | --- | --- |
| **1** | 99.70% | 0.30% | 0% |
| **2** | 0% | 99.70% | 0.30% |
| **3** | 19.90% | 7.20% | 72.90% |
| **4** | 1.90% | 24.70% | 73.40% |
| **5** | 12.50% | 12.10% | 75.50% |
| **6** | 14.50% | 6.50% | 79.00% |
| **7** | 2.30% | 16.50% | 81.20% |
| **8** | 5.20% | 12.10% | 82.70% |
| **9** | 2.70% | 14.20% | 83.10% |
| **10** | 2.60% | 13.60% | 83.80% |
| **11** | 10.10% | 5.00% | 84.90% |
| **12** | 10.00% | 5.00% | 85.00% |
| **13** | 4.80% | 8.30% | 86.90% |
| **14** | 5.70% | 6.80% | 87.50% |
| **15** | 2.00% | 10.50% | 87.60% |
| **16** | 2.60% | 9.40% | 88.10% |
| **17** | 3.80% | 8.00% | 88.20% |
| **18** |  | 11.10% | 88.90% |
| **19** | 4.20% | 6.70% | 89.20% |
| **20** | 4.80% | 4.80% | 90.50% |
| **21** | 7.20% | 2.30% | 90.50% |
| **22** | 2.10% | 7.40% | 90.60% |
| **23** | 2.40% | 7.10% | 90.60% |
| **24** | 7.20% | 2.20% | 90.70% |
| **25** | 3.80% | 5.20% | 91.00% |
| **26** | 4.20% | 4.70% | 91.00% |
| **27** | 4.40% | 4.40% | 91.20% |
| **28** | 7.90% | 0.30% | 91.80% |
| **29** | 5.10% | 2.80% | 92.00% |
| **30** | 4.40% | 3.50% | 92.10% |
| **31** | 5.60% | 2.30% | 92.10% |
| **32** | 2.00% | 5.90% | 92.20% |
| **33** | 4.40% | 3.30% | 92.30% |
| **34** | 4.00% | 3.60% | 92.40% |
| **35** | 2.50% | 5.00% | 92.50% |
| **36** | 4.80% | 2.70% | 92.50% |
| **37** | 6.40% | 0.90% | 92.70% |
| **38** | 4.50% | 2.50% | 93.00% |
| **39** | 6.50% | 0.50% | 93.10% |
| **40** | 6.40% | 0.60% | 93.10% |
| **41** | 3.20% | 3.60% | 93.20% |
| **42** | 3.30% | 3.20% | 93.50% |
| **43** | 6.50% |  | 93.50% |
| **44** | 3.60% | 2.90% | 93.50% |
| **45** | 4.20% | 2.20% | 93.70% |
| **46** | 5.10% | 1.20% | 93.70% |
| **47** | 2.50% | 3.60% | 93.90% |
| **48** | 0.80% | 5.30% | 93.90% |
| **49** | 5.40% | 0.70% | 93.90% |
| **50** | 2.30% | 3.80% | 93.90% |
| **51** | 3.50% | 2.50% | 94.00% |
| **52** | 0.90% | 5.10% | 94.00% |
| **53** | 2.50% | 3.40% | 94.10% |
| **54** | 3.60% | 2.30% | 94.10% |
| **55** | 5.70% | 0.20% | 94.10% |
| **56** | 2.50% | 3.40% | 94.10% |
| **57** | 5.60% | 0.20% | 94.20% |
| **58** | 4.90% | 0.80% | 94.30% |
| **59** | 1.30% | 4.40% | 94.30% |
| **60** | 5.20% | 0.40% | 94.40% |
| **61** | 4.40% | 1.10% | 94.50% |
| **62** | 3.80% | 1.60% | 94.50% |
| **63** | 1.80% | 3.70% | 94.50% |
| **64** | 5.40% |  | 94.60% |
| **65** | 4.40% | 1.00% | 94.60% |
| **66** | 1.80% | 3.50% | 94.70% |
| **67** | 3.40% | 1.80% | 94.70% |
| **68** | 5.00% | 0.30% | 94.70% |
| **69** | 4.80% | 0.50% | 94.70% |
| **70** | 2.60% | 2.50% | 94.80% |
| **71** | 3.90% | 1.20% | 94.80% |
| **72** | 3.00% | 2.10% | 94.80% |
| **73** | 1.80% | 3.30% | 94.90% |
| **74** | 2.60% | 2.40% | 94.90% |
| **75** | 1.80% | 3.30% | 94.90% |
| **76** | 2.90% | 2.00% | 95.00% |
| **77** | 4.00% | 1.00% | 95.00% |
| **78** | 3.70% | 1.20% | 95.00% |
| **79** | 1.80% | 3.10% | 95.10% |
| **80** | 1.90% | 2.90% | 95.20% |
| **81** | 1.70% | 3.10% | 95.20% |
| **82** | 4.20% | 0.40% | 95.40% |
| **83** | 3.30% | 1.20% | 95.50% |
| **84** | 4.50% |  | 95.50% |
| **85** | 3.70% | 0.80% | 95.50% |
| **86** | 4.30% | 0.20% | 95.50% |
| **87** | 2.50% | 2.00% | 95.50% |
| **88** | 1.80% | 2.70% | 95.50% |
| **89** | 2.00% | 2.40% | 95.60% |
| **90** | 3.40% | 0.90% | 95.70% |
| **91** | 4.30% |  | 95.70% |
| **92** | 1.60% | 2.70% | 95.70% |
| **93** | 3.10% | 1.20% | 95.80% |
| **94** | 0.30% | 3.80% | 95.80% |
| **95** | 1.50% | 2.60% | 95.80% |
| **96** | 1.10% | 3.00% | 95.80% |
| **97** | 3.50% | 0.60% | 95.90% |
| **98** | 2.70% | 1.40% | 95.90% |
| **99** | 0.50% | 3.50% | 96.00% |
| **100** | 2.40% | 1.60% | 96.00% |
| **101** | 3.70% | 0.20% | 96.10% |
| **102** | 3.50% | 0.30% | 96.20% |
| **103** | 2.90% | 1.00% | 96.20% |
| **104** | 3.80% |  | 96.20% |
| **105** | 2.40% | 1.40% | 96.20% |
| **106** | 2.70% | 1.10% | 96.20% |
| **107** | 1.90% | 1.70% | 96.40% |
| **108** | 3.50% |  | 96.50% |
| **109** | 2.80% | 0.70% | 96.50% |
| **110** | 2.40% | 1.00% | 96.50% |
| **111** | 0.90% | 2.60% | 96.50% |
| **112** | 1.20% | 2.30% | 96.50% |
| **113** | 2.80% | 0.80% | 96.50% |
| **114** | 2.10% | 1.40% | 96.50% |
| **115** | 3.00% | 0.40% | 96.60% |
| **116** | 1.50% | 2.00% | 96.60% |
| **117** | 2.60% | 0.70% | 96.60% |
| **118** | 0.40% | 3.00% | 96.60% |
| **119** | 1.90% | 1.40% | 96.70% |
| **120** | 2.90% | 0.40% | 96.70% |
| **121** | 3.30% |  | 96.70% |
| **122** | 3.20% |  | 96.80% |
| **123** | 2.70% | 0.40% | 96.90% |
| **124** | 2.10% | 0.90% | 96.90% |
| **125** | 1.60% | 1.60% | 96.90% |
| **126** | 2.40% | 0.70% | 96.90% |
| **127** | 1.80% | 1.40% | 96.90% |
| **128** | 2.70% | 0.20% | 97.00% |
| **129** | 3.00% |  | 97.00% |
| **130** | 3.00% |  | 97.00% |
| **131** | 2.60% | 0.40% | 97.00% |
| **132** | 1.40% | 1.50% | 97.10% |
| **133** | 2.80% |  | 97.20% |
| **134** | 0.50% | 2.30% | 97.30% |
| **135** | 1.20% | 1.40% | 97.40% |
| **136** | 2.20% | 0.30% | 97.40% |
| **137** | 1.10% | 1.40% | 97.50% |
| **138** | 0.30% | 2.20% | 97.50% |
| **139** | 1.70% | 0.80% | 97.50% |
| **140** | 1.60% | 0.80% | 97.50% |
| **141** | 0.60% | 1.80% | 97.60% |
| **142** | 2.00% | 0.40% | 97.60% |
| **143** | 2.30% |  | 97.70% |
| **144** | 1.30% | 1.10% | 97.70% |
| **145** | 1.20% | 1.00% | 97.80% |
| **146** | 1.60% | 0.50% | 97.80% |
| **147** | 0.30% | 2.00% | 97.80% |
| **148** | 1.90% | 0.20% | 97.80% |
| **149** | 1.80% | 0.30% | 98.00% |
| **150** | 1.70% | 0.20% | 98.00% |
| **151** | 0.50% | 1.50% | 98.00% |
| **152** | 1.70% | 0.20% | 98.00% |
| **153** | 1.40% | 0.50% | 98.10% |
| **154** | 1.30% | 0.50% | 98.20% |
| **155** | 1.30% | 0.40% | 98.20% |
| **156** |  | 1.40% | 98.60% |
| **157** | 1.30% |  | 98.70% |
| **158** | 1.30% |  | 98.80% |
| **159** | 1.10% |  | 98.90% |
| **160** | 0.70% | 0.30% | 99.00% |
| **161** |  | 0.90% | 99.10% |
| **162** | 0.90% |  | 99.10% |
| **163** | 0.50% | 0.50% | 99.10% |
| **164** | 0.70% |  | 99.30% |
| **165** | 0.30% | 0.30% | 99.30% |
| **166** | 0.60% |  | 99.40% |
| **167** |  | 0.20% | 99.80% |
| **168** |  |  | 100.00% |
| **169** |  |  | 100.00% |
|  |  |  |  |
| **ALL** | **3.50%** | **2.70%** | **93.80%** |
